# Identification of Candidate Partner Cells in Tumor–Stromal Interactions by Histology-Based Spatial Analysis for Prognostic Stratification After Neoadjuvant Chemotherapy

**DOI:** 10.3390/cancers18213450

**Published:** 2026-10-26

**Authors:** Naing Ye Aung, Takanobu Kabasawa, Yuki Hamada, Fuyuhiko Motoi, Mitsuru Futakuchi

**Affiliations:** 1 Department of Pathology, Yamagata University, Faculty of Medicine, 2-2-2 Iida-Nishi, Yamagata 990-9585, Japan; 2 Department of Surgery 1, Yamagata University, Faculty of Medicine, Yamagata 990-9585, Japan

**Keywords:** histology-based spatial analysis (HSA), proximity analysis, candidate partner cells, tumor–stromal interactions, tumor microenvironment (TME), neoadjuvant chemotherapy (NAC), prognostic stratification

## Abstract

Despite advances in multidisciplinary treatment, recurrence remains a major clinical challenge in refractory cancers. Many patients have residual tumor after neoadjuvant chemotherapy (NAC), and clinical outcomes among patients without a pathological complete response (non-pCR) are heterogeneous. The spatial relationships between residual tumor cells and surrounding stromal cells may provide information for further prognostic stratification. In this review, we discuss histology-based spatial analysis (HSA) of surgical specimens, with particular attention to pancreatic ductal adenocarcinoma. We describe four steps that connect existing biological, histological, and clinical findings. First, intercellular distances and local cell numbers are compared to identify candidate partner cells. Second, functional and molecular studies examine their interactions with tumor cells, including effects on survival and drug resistance. Third, HSA of immunostained patient specimens measures the number, size, and localization of foci containing cell relationships supported by these studies. Fourth, these measurements are evaluated in relation to recurrence and survival, alongside established clinicopathological factors. This approach uses archival surgical specimens to connect biological findings with patient outcomes. Its value for prognostic stratification after NAC requires validation in large clinical cohorts.

## Simple Summary

Patients with cancer remaining after chemotherapy given before surgery can have different outcomes. This review examines how the arrangement of cancer cells and surrounding non-cancerous cells in surgical specimens could help assess the risk of recurrence, with particular attention to pancreatic cancer. We review studies of cell interactions, tissue analysis, and patient outcomes. Distances and local cell numbers can be used to select cell populations for experimental study. Cell relationships supported by these experiments can then be examined in stained patient tissue, and the number, size, and location of the areas containing these relationships can be measured. Stored surgical specimens allow these measurements to be compared with recurrence and survival. Further studies in large patient groups are needed to determine whether this approach adds useful information for risk assessment.

## 1. Introduction

Neoadjuvant chemotherapy (NAC) is used for many solid tumors, and the efficacy of neoadjuvant therapy is commonly assessed histopathologically in resected surgical specimens after treatment. Pathological response is classified by the presence and extent of residual tumor, including pathological complete response (pCR) and non-pCR. The proportion of cases achieving a pCR varies markedly among cancer types.

In breast cancer, pCR has been reported in 33.6% of patients with triple-negative disease and 50.3% of those with hormone receptor-negative, HER2-positive disease treated with NAC plus trastuzumab [1]. Adding pertuzumab to trastuzumab-based NAC has also been shown to increase pCR rates in HER2-positive disease [2]. In contrast, pathological complete response is uncommon in pancreatic ductal adenocarcinoma (PDAC) after neoadjuvant therapy, and most resected specimens contain residual tumor [3]. Non-pCR cases remain at substantial risk of recurrence across tumor types, including breast and pancreatic cancers [4,5]. Because a substantial proportion of patients have non-pCR disease after NAC and their clinical outcomes are heterogeneous, further stratification of prognosis within this population is clinically important. This raises the question of whether residual tumor tissue can be used to further stratify non-pCR patients according to prognosis. Residual tumor tissue of non-pCR cases comprises biologically heterogeneous cell populations. These cellular populations are not randomly distributed but are spatially organized within the residual tumor microenvironment. In PDAC, for example, functionally distinct cancer-associated fibroblast (CAF) populations show different distributions relative to tumor cells [6,7]. These observations suggest that residual tumors after NAC represent spatially organized tumor microenvironments comprising both surviving tumor cells and stromal components. Spatial organization of the tumor microenvironment (TME) has also been associated with clinical outcomes in several cancers [8,9]. These findings support examining whether spatial information in post-NAC residual tumors can be used for further prognostic stratification.

Molecular pathological markers and omics analyses have characterized the molecular and genetic features of tumor cells and contributed substantially to the development of precision medicine. Indeed, molecular alterations involving HER2, EGFR, and ALK, together with PD-L1 expression, are used to select targeted therapies and immunotherapy [10]. Thus, conventional molecular pathological assessment has focused predominantly on tumor-cell-intrinsic features, while spatial information involving surrounding stromal components has been less extensively incorporated. This limitation is clinically relevant because tumors with similar histological or molecular profiles can exhibit markedly different therapeutic responses and clinical courses [11]. Accumulating evidence has shown that tumor invasion, therapeutic resistance, and prognosis are influenced not only by tumor-cell-intrinsic features but also by interactions with stromal components, including cancer-associated fibroblasts (CAFs), immune cells, and the extracellular matrix (ECM) [12,13]. In residual tumors after NAC, stromal components have been associated with treatment response and recurrence risk [14]. However, bulk omics requires tissue homogenization. Single-marker immunohistochemistry (IHC) preserves spatial information but has limited capacity to distinguish multiple phenotypically defined cell populations, making it difficult to directly evaluate their spatial relationships. Thus, the spatial relationships between tumor cells and surrounding stromal cell populations represent an additional dimension of tissue information that is not adequately captured by conventional tumor-cell-centered assessment.

Spatial analysis can address this gap by quantitatively assessing the relationships between defined target cells and surrounding stromal cells. By comparing intercellular distances and local cell abundance, cell populations showing preferential spatial relationships with target cells can be identified as candidate partner cells. To identify spatial relationships between tumor and stromal cells that are biologically relevant and associated with patient prognosis, spatial, functional, and clinical information need to be integrated. First, candidate partner cells can be identified based on their spatial relationships with defined target cells. Second, the biological relevance of these relationships can be assessed using functional evidence. Third, histology-based spatial analysis (HSA) of immunostained patient specimens can be used to examine the number, size, and localization of corresponding foci. Fourth, the clinical relevance of these measurements can be evaluated by examining associations with patient outcomes.

Recent advances in spatial biology have provided a variety of technologies for investigating the spatial organization of the tumor microenvironment (TME). High-plex spatial platforms, including multiplex immunohistochemistry (mIHC) [15], imaging mass cytometry (IMC) [16], CODEX [17], and spatial transcriptomics (ST) [18], enable the simultaneous visualization of multiple molecular markers or gene expression profiles while preserving their precise spatial organization within tissue. These technologies have enabled detailed characterization of cellular composition and spatial organization within the TME and have expanded the analysis of tumor–stromal relationships [9].

Recent studies have investigated the spatial organization of the tumor microenvironment (TME) from several complementary perspectives, including (1) neighborhood analysis focusing on spatial relationships between tumor and immune cells [19]; (2) analysis of the spatial organization of multiple cell populations [20,21]; and (3) artificial intelligence (AI)-based spatial analysis of routine histological images [22,23]. Together, these advances provide a basis for histology-based spatial analysis as a practical approach to evaluating spatial relationships in routine histological sections. Histology-based spatial analysis uses routine histological sections to quantitatively characterize the spatial relationships between tumor cells and surrounding stromal cell populations.

Li et al. defined 10 types of cellular neighborhoods from the arrangements of tumor, immune, and stromal cells in 41-plex CODEX data from 457 patients with non-small-cell lung cancer. They developed CANVAS to infer the corresponding spatial organization from hematoxylin and eosin (H & E)-stained images and applied it to more than 5000 patients across nine cancer types, examining associations with prognosis and outcomes after immunotherapy [23].

**Figure 1:**
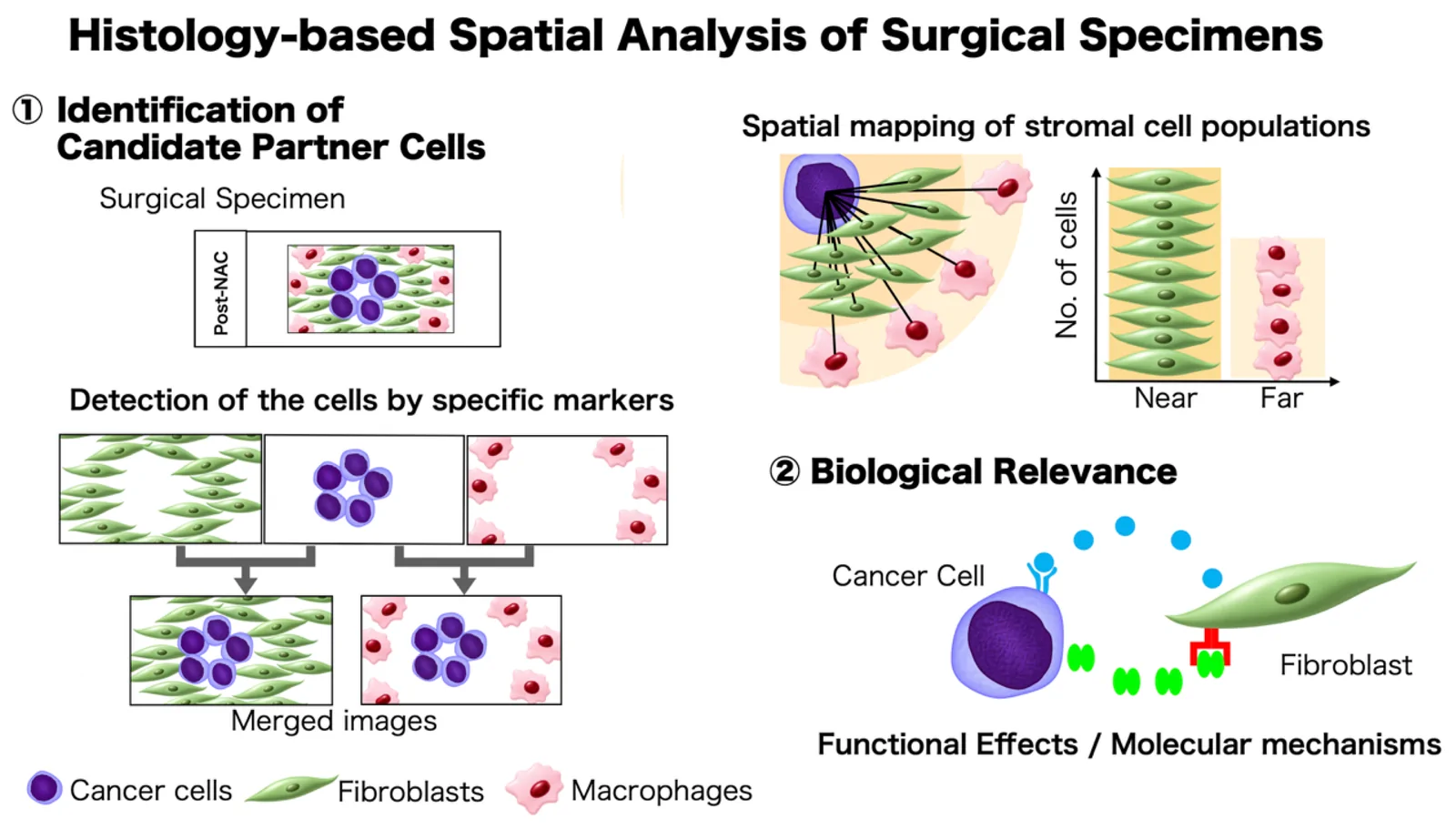
Identification of candidate partner cells and assessment of their biological relevance. Steps 1 and 2 of the proposed four-step framework are shown. In Step 1, tumor and stromal cell populations in post-NAC specimens are identified with specific markers. Cell maps from differently stained serial sections are registered and overlaid, and HSA compares intercellular distances and local cell numbers. Populations showing preferential relationships with defined tumor cells are selected as candidate partner cells. In Step 2, functional and molecular studies, including in vitro experiments, examine their interactions and effects on tumor-cell survival and drug resistance. Figure 2 shows the subsequent evaluation of corresponding foci in patient specimens and their prognostic relevance.

**Figure 2:**
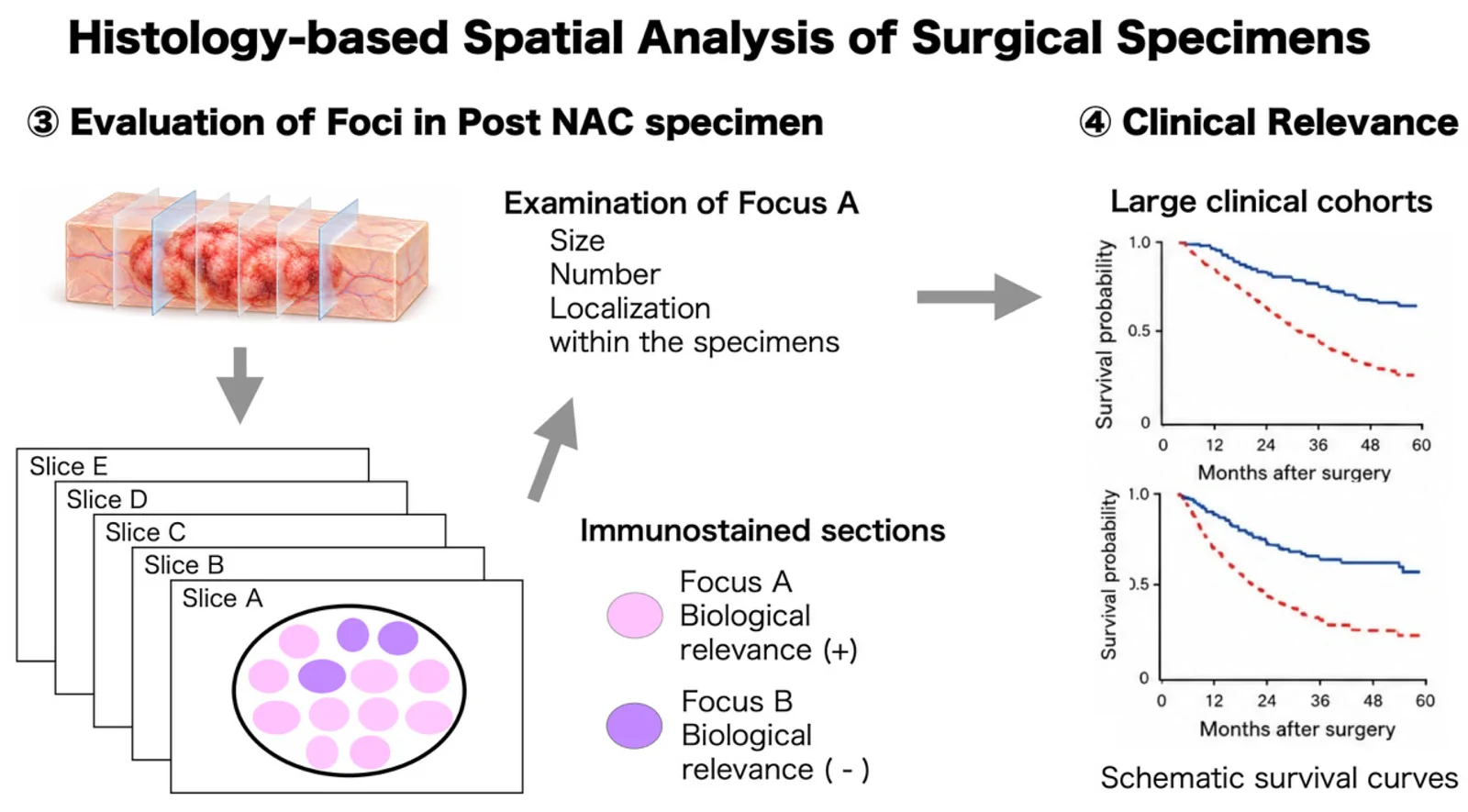
Evaluation of tumor–stromal interaction foci in post-NAC specimens and their clinical relevance. Steps 3 and 4 follow the biological assessment in Figure 1. In Step 3, HSA of immunostained sections identifies foci containing residual tumor cells and the defined stromal populations. Focus A (pink, +) represents cell relationships supported by biological evidence from Step 2; Focus B (purple, −) lacks that support. These labels indicate biological, not prognostic, relevance. The number and size of Focus A foci are measured. Their three-dimensional localization and distribution within the specimen are examined by relating sections to their gross sampling positions. In Step 4, patient-level measurements require testing in large cohorts for associations with recurrence and survival and potential value for prognostic prediction after NAC. The survival curves are schematic, not patient data.

This review examines the potential of histology-based spatial analysis of surgical specimens to identify candidate partner cells, assess the biological relevance of their spatial relationships, and support prognostic assessment after NAC. Section 2 reviews the histopathological and clinical basis for spatial analysis of tumor–stromal relationships. Section 3 and Section 4 describe the analytical principles and biological and clinical relevance of their spatial relationships, followed by future perspectives in Section 5. Figure 1 and Figure 2 summarize the proposed four-step framework.

In summary, patients with non-pCR disease after NAC have different outcomes, and the spatial organization of tumor and stromal cells has been associated with prognosis in several cancers. These findings support examining whether spatial information from residual tumors can further stratify patients with non-pCR disease according to prognosis. This review connects biological studies with the measurement of cell relationships and corresponding foci in patient tissue, and with subsequent evaluation against clinical outcomes.

## 2. Tumor–Stromal Interactions in Refractory Cancers

### 2.1. Spatial Associations Between Tumor and Stromal Cells at Sites of Tumor Progression

Histopathological studies of colorectal cancer have shown more aggressive features at the invasive front than in the superficial tumor region [24,25]. Here, the superficial tumor region is the portion facing the colonic lumen, and the invasive front is the leading edge invading deeper into the bowel wall.

For example, Kawamura et al. reported decreasing TOPO1 positivity with depth in 20 colorectal cancer cases: 80.3 ± 12.7% at Depth 1, 60.0 ± 28.8% at Depth 2, 30.4 ± 33.4% at Depth 3, 27.5 ± 32.6% at Depth 4, and 5.7 ± 6.7% at Depth 5. Depth 1 is the superficial mucosal region, whereas Depth 5 is the tumor budding region at the invasive front. The authors discussed low TOPO1 expression as potentially related to irinotecan resistance [26]. This illustrates a regional phenotypic difference that may be relevant to drug resistance.

During metastatic progression, early lesions initially consist mainly of proliferating tumor cells. As lesions grow, tumor-derived angiogenic factors induce neovascularization from the surrounding host tissue. Experimental and clinicopathological studies have described this process [27,28,29].

Tumor and stromal cells are frequently found in close spatial association at invasive fronts and peripheral regions [27,28,29]. Tumor-cell proliferative activity and phenotype vary within the same tumor, and interactions with surrounding stromal cells may contribute to these differences. Intratumoral heterogeneity can therefore be examined by relating regional tumor-cell properties to the spatial distribution of the surrounding stromal cells.

### 2.2. Tumor–Stromal Interactions and Their Clinical and Biological Relevance in PDAC

PDAC is a refractory cancer. Reviews published in 2018 and 2020 reported a five-year survival rate of approximately 10% [30,31]. Treatment with curative intent remains largely dependent on surgical resection, and even multidisciplinary treatment incorporating chemotherapy and radiotherapy has resulted in only modest improvements in patient survival [30]. Neoadjuvant chemotherapy (NAC) has increasingly been used for patients with resectable and borderline resectable PDAC [32,33,34,35].

Pathological complete response is uncommon after neoadjuvant therapy for PDAC, and most resected specimens contain residual tumor [3]. Patients with non-pCR disease remain at risk of early recurrence or distant metastasis [36]. Dense desmoplastic stroma surrounding tumor cells is a characteristic histological feature of PDAC [37].

Cancer-associated fibroblasts (CAFs) represent a major cellular component of this desmoplastic stroma [38,39].

CAFs are often close to pancreatic cancer cells, suggesting the potential for tumor–stromal interactions [7].

In resected PDAC, a higher activated stroma index, defined as the ratio of αSMA-stained area to collagen-stained area, was independently associated with poorer survival [40]. Prognostic associations may differ among CAF subsets [41]. Desmoplastic stroma has also been associated with progression in metastatic lesions [42], and CAF marker expression patterns have been associated with overall survival after resection [43]. These studies suggest that the abundance and composition of CAF-associated desmoplastic stroma may provide prognostic information in patients with PDAC.

Experimental studies have shown that CAFs affect malignant properties of pancreatic cancer cells [44]. In experimental models, IL-6 from CAFs and pancreatic stellate cells supports pancreatic cancer cell survival and promotes invasion [7,45]. Pancreatic stellate cells also induce epithelial–mesenchymal transition (EMT) and increase migration in co-cultured pancreatic cancer cells [46]. Furthermore, experimental studies have demonstrated that CAFs contribute to chemoresistance in pancreatic cancer cells through secreted factors and exosomes, resulting in reduced sensitivity to anticancer drugs [47,48].

Factors secreted by tumor cells can alter stromal-cell phenotypes. Öhlund et al. showed that conditioned medium from pancreatic cancer organoids increased IL-6 expression and decreased expression of Acta2, which encodes αSMA, in CAFs [7]. This demonstrates an effect of tumor-derived factors on CAF gene expression and properties.

As described above, close spatial associations between tumor and stromal cells are frequently observed at invasive fronts and peripheral regions of solid tumors, including colorectal cancers, suggesting a potential role of tumor–stromal interactions in the aggressive features observed in these regions. Together, the histopathological, clinical, and experimental findings described above support the involvement of PDAC–CAF interactions in the malignant behavior of PDAC. More broadly, tumor–stromal interactions have been associated with prognosis in various refractory cancers [12]. Therefore, quantitative evaluation of the spatial relationships between tumor and stromal cells in surgical specimens may provide information relevant to prognostic assessment after NAC.

In summary, CAFs and other stromal cells can affect tumor-cell survival, invasion, and drug resistance, while tumor-derived factors can alter CAF phenotypes. For example, Öhlund et al. showed that conditioned medium from pancreatic cancer organoids increased IL-6 expression and decreased expression of Acta2, which encodes αSMA, in CAFs [7].

In PDAC, stromal abundance, composition, and CAF marker expression have been associated with prognosis. Tumor–stromal interactions have also been linked to prognosis in other refractory cancers [12]. These findings support identifying which tumor–stromal cell relationships merit investigation in patient tissue. Section 3 describes how distances and local cell numbers can be compared to select these cell combinations.

## 3. Identification of Candidate Partner Cells by Histology-Based Spatial Analysis

### 3.1. Quantitative Assessment of Spatial Relationships Between Target and Candidate Partner Cells

The amount of a mediator depends on the abundance of mediator-producing cells, whereas its spatial range of action depends on the distance from these cells. For example, membrane-bound RANKL transduces signals through direct binding to its receptor, RANK. In this case, a greater number of RANKL-producing cells may result in stronger signaling. Thus, the strength of tumor–stromal interactions may depend, at least in part, on the number of cells involved. Some cell populations, such as regulatory T cells (Tregs), have important biological roles even in small numbers. Cell number alone therefore does not determine the importance of a population. With other conditions, including individual cell function, held constant, cell number may affect the magnitude of an interaction through the amount of factors supplied locally and the opportunities for contact with target cells. This supports quantifying cell numbers together with intercellular distances.

Soluble RANKL generated by proteolytic cleavage can act on RANK-expressing cells located at a distance from the RANKL-producing cells [49]. Thus, the spatial range of action differs between membrane-bound and soluble forms.

For soluble factors, the responding cell’s exposure depends on concentration as well as whether the factor reaches it. Francis and Palsson modeled diffusion from a single secreting cell. Their model relates the concentration distribution and effective signaling range to secretion and diffusion. With other conditions held constant, it predicts decreasing concentration with distance and a limited range over which the concentration required for a response is maintained [50].

Kopesky et al. provided an experimental example of a concentration-dependent response. They cultured human osteoclast precursors with M-CSF and varied the concentration of added RANKL. Osteoclast numbers increased over 0–66 ng/mL RANKL, with no further increase at higher concentrations. The response therefore depended on RANKL concentration rather than its presence alone [51].

Together, these theoretical and experimental findings suggest that distance between producing and responding cells may affect the response through local concentration, even when a soluble factor acts at a distance. Measuring distance together with the number of mediator-producing cells can therefore inform the study of cell interactions.

Carstens et al. studied tissue microarrays (TMAs) made from formalin-fixed, paraffin-embedded (FFPE) tissue from 132 patients with PDAC who had not received neoadjuvant therapy. Multiplex immunofluorescence identified tumor cells and T-cell populations. Ripley’s L function was used to assess T-cell distributions relative to tumor-cell nuclear centers. The area under the L-function curve over 0–20 µm measured infiltration near tumor cells. Greater cytotoxic T-cell infiltration within this range was associated with longer overall survival [21].

Saltz et al. used deep learning to map tumor-infiltrating lymphocytes (TILs) in 5202 H & E whole-slide images from 4759 patients across 13 cancer types in The Cancer Genome Atlas (TCGA). Images were divided into 50 × 50 µm patches. TIL spatial fraction was the number of TIL-positive patches divided by the total number of tissue-containing patches, not the number of individual lymphocytes. Spatial distributions and clustering differed among cancer types, immune subtypes, and tumor molecular subtypes [52].

Distances between target cells and candidate partner cells have also been quantified [53]. Combining distances with local cell numbers characterizes their spatial relationships.

In colorectal cancer tumor budding areas, we examined CAFs and macrophages as potential partner cell populations. In this study, we focused on macrophages as candidate partner cells and quantitatively assessed both their number and distance from tumor cells [26]. This focus did not exclude CAFs as potential partner cells.

Kawamura et al. calculated the number of macrophages per tumor cell by dividing the macrophage count by the tumor-cell count in the same region. They measured tumor-cell–macrophage distances and plotted macrophage counts in distance intervals. For regional comparisons, they selected 1 mm^2^ regions at the invasive front without tumor budding and in tumor budding areas [26].

In HSA using HALO, serial sections stained with different immunohistochemical markers are digitized, and the positions of the cells of interest are detected in each section. Images are registered, and the cell positions are overlaid in a common coordinate system. Distances and local cell numbers can then be measured on this shared plane, rather than counting positive cells separately in each stain.

For example, Suzuki et al. overlaid PD-L1-positive cells and pan-cytokeratin-positive tumor cells on one coordinate map. They measured the distance from each PD-L1-positive cell to the nearest tumor cell and the number of surrounding PD-L1-positive cells per tumor cell in the analysis region [54].

Carstens et al. selected a 20 µm range to examine regions in which cell contact was likely [21]. This range is not a universal threshold. Depending on the question, candidate identification can compare cell distributions across distance intervals or count cells within a specified range. Distance ranges that correspond to biological findings or prognosis in post-NAC residual tumors require further investigation.

For post-NAC specimens, viable residual tumor cells and the stromal populations of interest should be identified using predefined histological and immunohistochemical criteria. Regions of interest (ROIs) should be defined before analysis. Measures include the distance to the nearest candidate cell and the number of candidate cells within a specified radius of each tumor cell. These measures should be interpreted in light of local cell density and tissue architecture, which can produce apparent proximity without a specific association. Spatial proximity alone does not establish a functional interaction; it selects candidates for molecular and functional evaluation. In this review, partner cells are cells that interact with tumor cells in a biologically relevant way. Candidate partner cells require further validation of the nature and biological relevance of their interactions before they are regarded as partner cells. Their effects may be either tumor-promoting or tumor-suppressing.

For these analyses, viable residual tumor should be located on H & E-stained sections, and ROIs should include the surrounding stromal cells of interest. Necrotic regions are excluded. Purely fibrotic regions without residual tumor cells are not selected, but fibrous stroma around viable tumor is retained when it contains the populations being studied.

H & E sections should be matched to the immunostained sections used for analysis. ROI boundaries and excluded regions should be recorded on the images. Selection and exclusion criteria should be specified before outcome analysis, and their reproducibility should be examined.

Section selection and ROI selection within sections are separate decisions. Carstens et al. reviewed H & E sections from untreated PDAC, selected two representative 1-mm-diameter tumor cores per patient for TMAs, and analyzed 5-µm-thick sections [21]. Saltz et al. used diagnostic H & E whole-slide images from TCGA and, when more than one diagnostic slide was available for a patient, used the slide designated DX1 for association analyses [52]. Post-NAC studies need to specify sampling sites, section numbers, the location, area, and number of ROIs, and how measurements are summarized for each patient before analyzing outcomes.

A two-dimensional section represents the analyzed region and may not show where foci are concentrated within the submitted organ. When focus number and size are used as patient-level measurements, each section’s gross sampling position should be recorded. This allows the distribution of foci within the organ, and how well selected sections represent it, to be investigated.

### 3.2. Identification of Candidate Partner Cells by Comparative Spatial Analysis

Subpopulations within a candidate partner cell population do not necessarily interact with target cells in the same manner. For example, macrophage populations can exhibit distinct activation states, historically described by the M1/M2 framework, with different functions in the TME [55,56]. Proximity analysis can quantify the spatial relationship of each subpopulation with target cells. We therefore considered that comparing these spatial relationships could identify subpopulations showing preferential spatial associations with target cells as candidate partner cells. Similarly, comparing the spatial relationships of multiple candidate cell types with target cells could identify those showing preferential spatial relationships with target cells. The same comparison can be made across different candidate cell types.

We applied this approach to the marginal region of triple-negative breast cancer (TNBC). We found that PD-L1+ stromal cells accumulated in the marginal region, and the majority of these cells were CD68+ macrophages. In contrast, CD68+ macrophages were diffusely distributed in both the marginal and core regions, so the overall distribution of CD68+ macrophages did not account for the preferential accumulation of PD-L1+ stromal cells in the marginal region. We therefore focused on the PD-L1-expressing subpopulation within the CD68+ macrophage population. To determine whether this subpopulation showed a specific spatial relationship with TNBC cells, we measured distances from PD-L1+ cells to their nearest TNBC cells and counted PD-L1+ cells per TNBC cell. This spatial analysis suggested that the PD-L1+ subpopulation, rather than the overall CD68+ macrophage population, may represent a candidate partner cell population for TNBC cells [54].

In the mandibular bone-invasive region of oral squamous cell carcinoma (OSCC), histological examination showed a layered stromal region interposed between OSCC and the mandibular bone, in which CAFs were enriched. We therefore focused on CAFs within this stromal region and evaluated their proximity to OSCC cells and osteoclasts. As a result, large numbers of CAFs were found in close proximity to both OSCC cells and osteoclasts. These findings suggested spatial relationships between CAFs and both OSCC cells and osteoclasts that may reflect their respective cellular interactions [57]. Within this layer, one CAF population was close to OSCC cells and another was close to osteoclasts, suggesting distinct spatial relationships with the respective cell types. This observation led us to consider that proximity analysis could distinguish spatially distinct subpopulations within a morphologically defined stromal population and identify candidate partner cells for a defined target cell population. Furthermore, if a specific stromal cell subpopulation can be identified by IHC or in situ hybridization (ISH) using phenotype-specific markers, candidate partner cells for this subpopulation could be identified by proximity analysis.

In summary, intercellular distances and local cell numbers can identify candidate partner cells by comparing the spatial distributions of populations and subpopulations. These comparisons help identify which stromal cell populations to examine for interactions with tumor cells, and where these populations are located within the tissue. Proximity alone does not establish biological relevance. In vitro and other experimental studies are therefore needed to determine the interactions involved and their effects on tumor-cell properties.

## 4. Biological and Clinical Relevance of Tumor–Stromal Spatial Relationships

### 4.1. Prognostically Relevant Regions in Post-NAC PDAC

Spatial analysis can identify candidate partner cells, but its additional prognostic value in residual PDAC after NAC remains to be established. The next sections examine how biological findings can guide focus measurements in patient tissue and their subsequent clinical evaluation.

Most resected PDAC specimens contain residual tumor after neoadjuvant therapy, and patients with non-pCR disease remain at high risk of recurrence [3,36].

Distinguishing outcomes within this group is an important clinical question.

Okubo et al. investigated histological features associated with prognosis using H & E-stained sections from surgical specimens of PDAC after NAC. They defined the Area of Residual Tumor (ART) as the sum of the areas occupied by multiple viable residual tumor regions and demonstrated that ART was an independent predictor of recurrence-free survival (RFS) in post-NAC PDAC. These findings suggest that viable residual tumor regions identified in routine H & E-stained sections represent histological regions relevant to prognostic assessment after NAC [58].

They digitized H & E sections from the largest cross-section containing residual tumor and outlined viable tumor areas. Necrotic tumor cells, fibrosis, in situ lesions, and acellular mucin pools were excluded. Viable foci more than 2 mm from the largest tumor area were measured separately, and their areas were summed to obtain ART [58].

Clinical validation should include patients with residual PDAC who undergo resection after neoadjuvant chemotherapy. Before analyzing outcomes, investigators should specify the criteria for identifying candidate CAF populations, the regions to be examined, how measurements of the number, size, and localization of foci are summarized per patient, and the primary outcome, such as RFS. Clinicopathological variables should also be prespecified, with pathological stage, ART, pathological treatment response, and lymph node status considered as candidates. Multivariable Cox regression can then test whether focus number and size are associated with outcomes after accounting for the selected factors. Added prognostic value needs to be assessed beyond these factors, and findings need to be validated in an independent cohort. Variation in staining, image analysis, and ROI selection also requires assessment.

### 4.2. Biological Relevance of Spatial Relationships Between Tumor Cells and CAFs

CAFs contribute to pancreatic cancer aggressiveness [37,41,44]. Given the prominent desmoplastic reaction observed histologically in pancreatic cancer, CAFs are an important stromal cell population to examine in relation to tumor cells. However, spatial proximity between tumor cells and CAFs alone does not necessarily indicate the biological relevance of their interaction. Furthermore, CAFs are known to exhibit multiple phenotypes [6,7]. Therefore, proximity analysis can be used to identify CAF phenotypes showing preferential spatial relationships with tumor cells as candidate partner cells. In post-NAC surgical specimens, functional evidence can then be integrated with these spatial relationships to assess the biological relevance of candidate tumor–stromal interactions. Distinguishing CAF phenotypes has limited value if the sole purpose is to confirm that CAFs are close to tumor cells. The relevant question is whether the different phenotypes are associated with different biological effects. Comparing their distances from tumor cells and local cell numbers can identify which phenotype occurs near which tumor cells, and in what numbers. This selects candidate partner cells for functional studies; the effects and biological relevance of each interaction still require validation. Reported phenotypes include myofibroblastic CAFs (myCAFs), inflammatory CAFs (iCAFs), and antigen-presenting CAFs (apCAFs), characterized by markers such as αSMA, IL-6, and MHC class II, respectively [6,7,38].

However, expression of a single marker does not, by itself, establish that a cell belongs to a particular CAF phenotype. Candidate CAF populations should first be characterized by high-dimensional profiling or multiplex-marker analysis.

A smaller validated marker panel could then be selected for use with archival histological sections. After confirming the reproducibility of cell identification and spatial measurements, proximity analysis could assess the relationships between these marker-characterized CAF populations and viable residual tumor cells.

CAF phenotypes defined by mRNA expression may be identified in sections by ISH for distinguishing transcripts. Phosphorylation of downstream signaling molecules may also help identify CAFs responding to factors secreted by tumor cells.

Once these methods of cell identification are validated, distances from tumor cells and local cell numbers can be measured to identify candidate partner cells. Such studies may extend proximity analysis to additional CAF populations.

Functional studies are then needed to determine whether candidate CAF populations affect tumor-cell survival, aggressiveness, or treatment resistance [7,47,48].

The same approach could be applied to newly identified CAF subpopulations, provided that they can be reliably identified in archival specimens and their spatial relationships with residual tumor cells can be measured reproducibly [6].

These newly identified subpopulations need to be evaluated, as with established CAF phenotypes, to determine whether they can serve as candidate partner cells, whether their interactions with tumor cells are biologically relevant, and whether regions containing these interactions are associated with patient prognosis. Proximity analysis can be used to quantify the spatial relationships between newly identified CAF subpopulations and tumor cells. Based on these spatial relationships, CAF subpopulations that may serve as candidate partner cells can be identified.

Fibrosis, necrosis, acellular mucin, and inflammation after NAC can alter tissue architecture, cell density, and intercellular distances. Proximity in such tissue does not by itself identify a prognostically relevant focus.

The proposed four-step approach separates HSA-based candidate identification (Step 1), biological analysis (Step 2), examination of corresponding foci in patient tissue (Step 3), and clinical evaluation (Step 4).

In Step 3, HSA of immunostained sections examines residual tumor cells together with stromal populations regarded as partner cells on the basis of biological evidence, such as effects on survival or drug resistance. The number, size, and localization of foci containing the corresponding cell relationships are measured in patient tissue.

Focus A in Figure 2 denotes foci selected on the basis of the biological findings in Step 2. It does not denote an established association with patient prognosis.

Step 3 also examines the three-dimensional localization and distribution of foci within the submitted organ. Future studies need to sample the entire submitted organ, prepare histological sections, and record each section’s gross sampling position. HSA can then examine where foci occur and how their number and size differ among regions.

These distributions can inform section and ROI selection. Further work needs to establish how well a limited set of sections represents the organ-level distribution of foci.

In Step 4, focus measurements for each patient are related to recurrence and survival after NAC. Their prognostic value requires evaluation in large clinical cohorts (Figure 2).

Thus, HSA serves two different purposes: selecting candidate cell populations before biological analysis and measuring corresponding foci in patient tissue afterward. The latter measurements connect biological findings with clinical evaluation.

### 4.3. Clinical Validation of Spatial Relationships in Large Cohorts

The four-step approach uses complementary spatial methods. Detailed cell characterization can guide selection of populations for biological study, while HSA of archival sections can measure corresponding foci for subsequent clinical evaluation in non-pCR PDAC.

High-plex platforms include mIHC and multiplex immunofluorescence (mIF) [15], IMC [16], CODEX [17], and ST [18]. These platforms enable detailed molecular mapping of the TME, facilitating the identification of previously unrecognized subpopulations and investigation of their molecular features. In contrast, their application to large retrospective cohorts is constrained by cost, specialized instrumentation, and specimen requirements. Table 1 compares these high-plex platforms with proximity analysis based on routine histological specimens.

Proximity analysis of routine H & E or single-marker IHC sections can measure distances and distributions for cell populations identifiable by those stains. Specific CAF subpopulations are difficult to identify this way. Their distinguishing markers and identification by IHC or ISH require validation before proximity analysis. Archival material from routine pathology allows these measurements to be evaluated in large retrospective cohorts. High-plex characterization and HSA-based focus measurements can therefore support different parts of the biological and clinical investigation.

In summary, previous studies have described different CAF phenotypes and associations between histological findings in residual tumors and patient prognosis. Biological studies are needed to examine how candidate CAF populations affect tumor cells. HSA of immunostained sections can then identify foci containing the cell relationships supported by these studies and measure their number, size, and localization. Using archival surgical specimens, these measurements can be related to recurrence and survival in large clinical cohorts. Further studies are needed to determine whether they provide additional prognostic information beyond established clinicopathological factors after NAC.

## 5. Conclusions and Future Directions

We reviewed the potential of histology-based spatial analysis of surgical specimens to support prognostic assessment after NAC. Clinical application depends on four linked steps: identifying candidate partner cells, characterizing their biological effects and mechanisms of interaction with tumor cells, examining the number, size, and localization of corresponding foci in patient specimens, and validating the prognostic relevance of these measurements.

### 5.1. Mechanistic Characterization of Candidate Spatial Interactions

Proximity analysis can identify candidate cell populations that may interact with a defined target population. Integrating the spatial relationships between these candidate and target cell populations with spatial transcriptomic or proteomic data may help clarify the molecular basis and biological significance of their potential interactions.

### 5.2. Clinical Validation and Prognostic Modeling for Patient Stratification

In large retrospective cohorts, HSA-derived measurements of the number, size, and localization of foci in archival specimens can be linked to long-term clinical outcomes to evaluate the prognostic relevance of the corresponding tumor–stromal interactions. Spatial interactions supported by both biological and clinical evidence could then inform patient stratification and the development of prognostic models.

### 5.3. Conclusions

Histology-based spatial analysis provides a sequential framework for identifying candidate partner cells, assessing the biological relevance of their spatial relationships, quantifying corresponding foci in patient specimens, and evaluating associations between these measurements and clinical outcomes. Applied to surgical specimens from patients with known outcomes, this framework may support prognostic stratification after NAC. By linking molecular and functional findings derived from surgical specimens to clinical outcome data for the patients from whom the specimens were obtained, histology-based spatial analysis may bridge mechanistic investigation and prognostic assessment after NAC.

## Figures and Tables

**Table 1:** Comparison of representative spatial biology platforms and routine histology-based proxiConfirmedmity analysis.

Technology	Molecular Depth	Spatial Resolution	Accessibility/Scalability	Typical Application
mIHC/mIF [15]	10–40 proteins	Single-cell	Moderate cost; requires validated antibody panels	Phenotyping immune–stromal interfaces
Imaging Mass Cytometry (IMC) [16]	30–60 proteins	Subcellular (≈1 µm)	High cost; low throughput	Multiplex profiling of tumor microregions
CODEX [17]	40–100 proteins	Subcellular	Complex instrumentation; limited cohort size	Immune landscape mapping
Spatial Transcriptomics (ST) [18]	Thousands of genes	50–100 µm (spot based)	High cost	Gene-expression mapping with spatial context
**Histology-based Spatial Analysis (HSA)**	5–10 markers	Single-cell	Uses routine FFPE slides; scalable to hundreds of cases	Quantitative analysis of tumor–stroma and immune interactions

Data summarized from Refs. [15,16,17,18]. “Accessibility/Scalability” refers to feasibility for large retrospective or clinical cohort studies. Abbreviations: mIHC, multiplex immunohistochemistry; mIF, multiplex immunofluorescence; IMC, Imaging Mass Cytometry; ST, Spatial Transcriptomics; HSA, Histology-based Spatial Analysis.

## Data Availability

No new data were created or analyzed in this study. Data sharing is not applicable to this article.
